# Usual source of care and geographic region are largest predictors of healthcare quality for incident lupus nephritis in US Medicaid recipients

**DOI:** 10.1186/ar3951

**Published:** 2012-09-27

**Authors:** J Yazdany, C Feldman, J Liu, MM Ward, MA Fischer, KH Costenbader

**Affiliations:** 1 University of California, San Francisco, CA, USA; 2 Brigham and Women's Hospital, Boston, MA, USA; 3 National Institute of Arthritis and Musculoskeletal and Skin Diseases, Bethesda, MD, USA

## Background

Little is known about the quality of healthcare delivered to patients with lupus nephritis in the United States and the major determinants of quality remain unknown. We aimed to examine the sociodemographic, geographic, and healthcare system factors associated with performance on a healthcare quality measure in a nationwide cohort of Medicaid recipients with incident lupus nephritis.

## Methods

We used US Medicaid analytic extract (MAX) data from 2000 to 2004 containing person-level files on Medicaid eligibility, utilization and payments. We identified patients meeting a validated administrative data definition of incident lupus nephritis, and used this group as the denominator population for the quality metric (QM). The QM numerator assessed receipt of **i**nduction therapy with glucocorticoids and another immunosuppressant (azathioprine, mycophenolate mofetil, mycophenolic acid, cyclophosphamide, cyclosporine A, or tacrolimus) within 90 days of lupus nephritis onset. Patients with end-stage renal disease were excluded. We used multivariable logistic regression models to examine sociodemographic (age, sex, race/ethnicity), geographic (US region), and healthcare (health professional shortage areas, HPSAs, from the Area Resource File) predictors of higher performance. We also examined the restrictiveness of Medicaid benefits in each state, defined by less generous medication coverage policies (mandatory generic substitution, requirements for prior authorization and drug co-payments), and whether the patient's usual source of care was the emergency department or the ambulatory setting (>50% visits).

## Results

A total of 974 Medicaid recipients met the definition of incident lupus nephritis. The mean age was 39 years (SD 12), 93% were female, and most were African American (African American 48%, White 27%, Hispanic 13%, Asian 6%). Individuals were geographically dispersed (20% Midwest, 22% Northeast, 34% South, 24% West), and 95% resided in partial or complete HPSAs. One hundred and sixty-four individuals resided in states with more restrictive Medicaid benefits. At 90 days, only 16% of patients met all numerator components of QM1; 45% of individuals received only steroids (mean prednisone dose 28 mg/day), and 3% received an immunosuppressant alone. Among those treated with an immunosuppressant, 31% received azathioprine, 47% received mycophenolate, 14% received cyclophosphamide, and 11% received a calcineurin inhibitor. For 20% (*n *= 192) of patients, the usual source of care was in the emergency setting. In multivariable logistic regression models, younger individuals were more likely to receive optimal treatment (OR for 18 to 34 years vs. 51 to 64 years = 3.5, CI = 1.6 to 7.6), while those living in the South and Midwest were less likely (OR = 0.49, CI = 0.24 to 0.67 and OR = 0.30 CI = 0.15 to 0.61, respectively). Those whose usual source of care was the emergency department were less likely to receive optimal treatment (OR = 0.47, CI = 0.28 to 0.81). In this adjusted analysis, we did not find significant associations for race/ethnicity, HPSA or Medicaid restrictiveness with QM performance.

## Conclusion

Most US Medicaid recipients with incident lupus nephritis in our study did not receive timely induction therapy, and many were treated with high-dose steroids alone. We found significant geographic variation in performance, with the South and Midwest having lower performance than other regions. A substantial number of Medicaid patients with lupus nephritis used the emergency department as a usual source of care and performance on the QM is lower in this setting. These data suggest a need for targeted quality improvement interventions, including increasing access to appropriate ambulatory care for Medicaid recipients with lupus nephritis.

